# Phosphotriesterase-related protein sensed albuminuria and conferred renal tubular cell activation in membranous nephropathy

**DOI:** 10.1186/1423-0127-21-32

**Published:** 2014-04-22

**Authors:** Chao-Wen Cheng, Li-Chien Chang, Tzu-Ling Tseng, Chia-Chao Wu, Yuh-Feng Lin, Jin-Shuen Chen

**Affiliations:** 1 Graduate Institute of Clinical Medicine, College of Medicine, Taipei Medical University, Taipei, Taiwan; 2 Center for Translational Medicine, College of Medical Science and Technology, Taipei Medical University, Taipei, Taiwan; 3 Graduate Institute of Medical Sciences, Taipei, Taiwan; 4 School of Pharmacy, National Defense Medical Center, Taipei, Taiwan; 5 Biomedical Technology & Device Research Laboratories, Industrial Technology Research Institute, Hsinchu, Taiwan; 6 Division of Nephrology, Department of Internal Medicine, Tri-Service General Hospital, 325, Sec. 2, Cheng-Kung Rd., Neihu 114 Taipei, Taiwan, Republic of China; 7 Department of Internal Medicine, Shuang Ho Hospital, Taipei Medical University, New Taipei, Taiwan

**Keywords:** Albuminuria, Nephrotic syndrome, Phosphotriesterase-related protein (PTER)

## Abstract

**Background:**

Membranous nephropathy (MN) is a common cause of nephrotic syndrome that may progress to end-stage renal disease (ESRD). The formation of MN involves the *in situ* formation of subepithelial immune deposits and leads to albuminuria; however, the underlying mechanism of how MN leads to ESRD remains unclear. The aim of this study was to investigate the expression and biological functions of phosphotriesterase-related protein (PTER) in MN.

**Results:**

In the progression of MN, the expression of PTER increased significantly and was mainly expressed in the renal tubular cells. Both mRNA and protein expression levels of PTER were increased in a concentration- and time-dependent manner in the *in vitro* albuminuria tubular cell model. Silencing the expression of PTER by RNA interference diminished albuminuria-induced inflammatory and pro-fibrotic cytokines production.

**Conclusions:**

Our findings reveal that PTER may sense albuminuria in the progression of MN, induce tubular cell activation and lead to ESRD.

## Background

Membranous nephropathy is a leading glomerular disease and represents one of the most frequent causes of adult idiopathic nephrotic syndrome, with up to 40-50% of patients progressing to end-stage renal disease (ESRD) [1]. The definition of MN is the presence of immune complexes depositing along the glomerular basement membrane (GBM) in the sub-epithelial location [2]. The subsequent expansion of the GBM results in a membrane-like thickening of the glomerular capillary wall and causes the characteristic changes of albuminuria [3].

The formation of immune complex deposition may be mediated by circulating autoantibodies which bind to endogenous antigens *in situ* on the podocyte foot process, which in turn react with glomerular endothelial cells and GBM [4]. The M-type phospholipase A2 receptor (PLA2R) has recently been identified as the first major human antigenic target in adults MN [5]. In addition to endogenous antigens, immune complexes may also be formed by the reaction of antibodies to exogenous antigens, for example cationic antigens deposit in the sub-epithelial space, and cause membrane damage, leading to albuminuria [6,7]. The renal functional outcome for patients with chronic glomerulopathy can be predicted histologically by the severity of chronic extraglomerular damage, such as peritubular capillary loss, tubular atrophy and interstitial fibrosis [8]. It has been suggested that chronic glomerular disease patients with high-grade albuminuria are more likely to develop chronic renal failure than patients with low-grade or no albuminuria [9]. In addition, urinary proteins themselves may elicit pro-inflammatory and pro-fibrotic effects that directly contribute to chronic tubulointerstitial damage. These phenomena suggest the albuminuria may also exacerbate the severity of glomerular damage [8]. In our previous study, we found that in the process of the kidney injury, certain intrinsic factors, such as vascular endothelial cell growth factor (VEGF), thrombospondin-1 (TSP-1) and plasminogen kringle domain 5 (K5), can be regulated and present the protective and pathogenic functions [10]. However, the underlying mechanism of intrinsic factors that will induce MN injury, leading to ESRD, remains largely unknown. Examining the differential renal mRNA and protein expression levels in the progression of disease may help to verify specific disease markers, and help us understand the pathogenesis of MN.

During the course of our pilot studies on both cDNA microarray and isobaric tag for relative and absolute quantification (iTRAQ) analysis in MN mouse model, we had observed both the mRNA and protein levels of phosphotriesterase-related protein (PTER) were up-regulated in the renal specimens of MN. PTER had been reported its expression in the kidney proximal tubular cells, and showed abnormal expression in injured and cystic kidneys [11]. Accordingly, we postulated PTER may play a crucial role in the pathophysiology of MN and its expression might be a useful predictive biomarker for the progression of MN.

## Methods

### Animal models

Animal studies were performed in accordance with institutional guidelines. Female BALB/c mice were used and separated into experimental and control groups. Experimental MN was induced by cationic bovine serum albumin (cBSA) as previously described [10]; however, the control group was not injected. Once urine dipsticks showed a marked albuminuria (++++), it was considered full-blown MN. Mice were then euthanized, and blood, urine and kidney samples were collected.

### Human GN

The institutional review board at the National Defense Medical Center (Taipei, Taiwan) approved the study. All patients were over 20 years old and capable of providing informed consent. In brief, patients with severe albuminuria were recruited for this study. These patients were required to have a kidney biopsy. Cases of MN, Focal segmental glomerulosclerosis (FSGS) and diabetic nephropathy (DN) were collected to confirm the experimental findings for the MN mouse model. Kidney tissues were obtained when patients underwent routine renal biopsies.

### Immunohistochemistry

The paraformaldehyde-fixed renal tissues were embedded in paraffin. Three micrometer sections were obtained, removed paraffin from sections and followed by rehydration. Immunohistochemical staining was performed by using the avidin-biotin immunoperoxidase method. After quenching the endogenous peroxidase activity and blocking with 1% BSA, the rabbit polyclonal anti-PTER antibody (GTX102860, GeneTex, San Antonio, TX, USA) was added and incubated at 4°C overnight. After washing, sections were incubated with biotinylated secondary antibody (Vector laboratories, Burlingame, CA, USA) for 40 mins, and treated with VECTASTAIN ABC (Vector laboratories) working solution for 30 mins. The reaction was then visualized by use of DAB chromogen. The developed tissues were counterstained with hematoxylin. Sections were then observed with an optical photomicroscope. Negative control was done by omitting the primary antibody.

### Western blot analysis

After mice were euthanized, the renal cortical region was collected for protein extraction. The protein concentration was determined by the BCA protein assay. Thirty microgram of protein from each samples were separated by sodium dodecyl sulfate-polyacrylamide gel electrophoresis. The gel was equilibrated in transfer buffer at room temperature, and the proteins were transferred onto polyvinylidene fluoride membranes (Immobilon-P, Millipore, Bedford, MA, USA) for 2.5 h at 4°C in transfer buffer. The membranes were then blocked with 2% BSA in TBST for 1 h at 37°C, washed with TBST, and incubated with anti-PTER (sc-107075, Santa Cruz Biotechnology, Santa Cruz, Ca, USA) or anti-â-actin antibodies at 4°C overnight. The next day, the membranes were then incubated with appropriated secondary antibodies rabbit anti goat for 1 h at room temperature. Bindings were visualized with the Western Lightning Chemiluminescence Reagent *Plus* (Perkin-Elmer Life Sciences, Boston, MA, USA) and the density of each band was determined. All the results were normalized for the amount of β-actin.

### *In vitro* albuminuria model

HK-2 cell (human kidney-2) is an immortalized proximal tubular epithelial cell line generated from a normal adult human kidney. It was cultured in DMEM/F12 medium supplemented with 5% FBS, 100U/ml penicillin, 100 μg/ml streptomycin, 2 mM L-glutamine, 5 μg/ml transferring and 5 ng/ml sodium selenite, 5 pg/ml T3, 5 ng/ml hydrocortisone, 5 pg/ml PGE1, and 10 ng/ml epidermal growth factor. HK-2 cells were incubated at the indicated concentrations of human serum albumin (HSA) and γ-globulin in serum and growth factor-free basal medium for 24 h and 48 h. The cells were harvested for western blot and RT-PCR analysis.

### Reverse-transcriptase polymerase chain reaction (RT-PCR)

Total RNA was extracted with Trizol reagent (Invitrogen Corporation) from the kidney cortex. Chloroform was added and the RNA was precipitated from the aqueous phase with isopropanol at 4°C. RNA was reconstituted in RNase-free water, and 18S and 28S rRNA were checked to confirm the integrity of the RNA extracted. Maxime RT-PCR PreMix Kit (Intron, Korea) was used for cDNA synthesis. Quantitative real-time PCR was performed using a FastStart Universal SYBR Green Master (Rox) reagent (Roche Diagnostics GmbH, Germany) according to the manufacturer’s protocol, and the results were analyzed using a LightCycler® 480 Real-Time PCR System.

The primers used in this study were as follows: PTER, forward 5′- GGAGTTTGCTCAACTTGGCTGC-3′ and reverse 5′- TCTTCACAGCCCTCTTCCACCA-3′; RANTES, forward 5′- CCTGCTGCTTTGCCTACATTGC-3′ and reverse 5′- ACACACTTGGCGGTTCTTTCGG-3′; MCP-1, forward 5′- AGAATCACCAGCAGCAAGTGTCC-3′ and reverse 5′- CAGCAAGAGCACAAGAGGAAG-3′. The housekeeping gene GAPDH (forward 5′- CAGCAAGAGCACAAGAGGAAG-3′ and 5′- TGGTACATGACAAGGTGCGG -3′) was used as the internal standard.

### siRNA Transfection

HK-2 cells (1 × 10^5^/well) were maintained in medium in 6-well plates. Once the cells were sub-confluent, the medium was changed to Transfection Medium (sc-36868, Santa Cruz), Transfection Reagent (TR, sc-29528, Santa Cruz) with PTER siRNA suspension (sc-90527, Santa Cruz) according to manufacturer’s instructions. The volume of TR was fixed at 4ul per the instructions. The optimal siRNA amount was determined by HK-2 transfecting with different volumes of PTER 2 and 4ul per well. After 24 h, cells from these different mixes were harvested for RNA extraction. RT-PCR was used to analyze the expression of PTER. Optimal ratio between the volume of TR and siRNA (TR-to-siRNA) was established based on when the knock-down efficacy of mRNA level reached higher than 80%.

Once the optimal ratio of TR-to-siRNA was determined, the subsequent experiments were performed as follows. First, HK-2 cells were treated with TR containing PTER siRNA, scramble siRNA (scRNA, sc-37007, Santa Cruz), and TR only. After 16 hr, the culture medium was replaced with fresh medium containing 1 mM HSA for 24 h, and then RNA was extracted. RT-PCR was used to analyze the gene expression levels.

### Statistical analysis

All results were presented as means ± standard deviation. Comparisons between two groups were analyzed by un-pair t-test. Differences among multiple groups were determined by the one-way analysis of variance (ANOVA) using Tukey’s method for *post hoc* analysis. *P < 0.05* was considered as statistically different.

## Results

### Differential renal tubular PTER expression in MN

**Figure 1:**
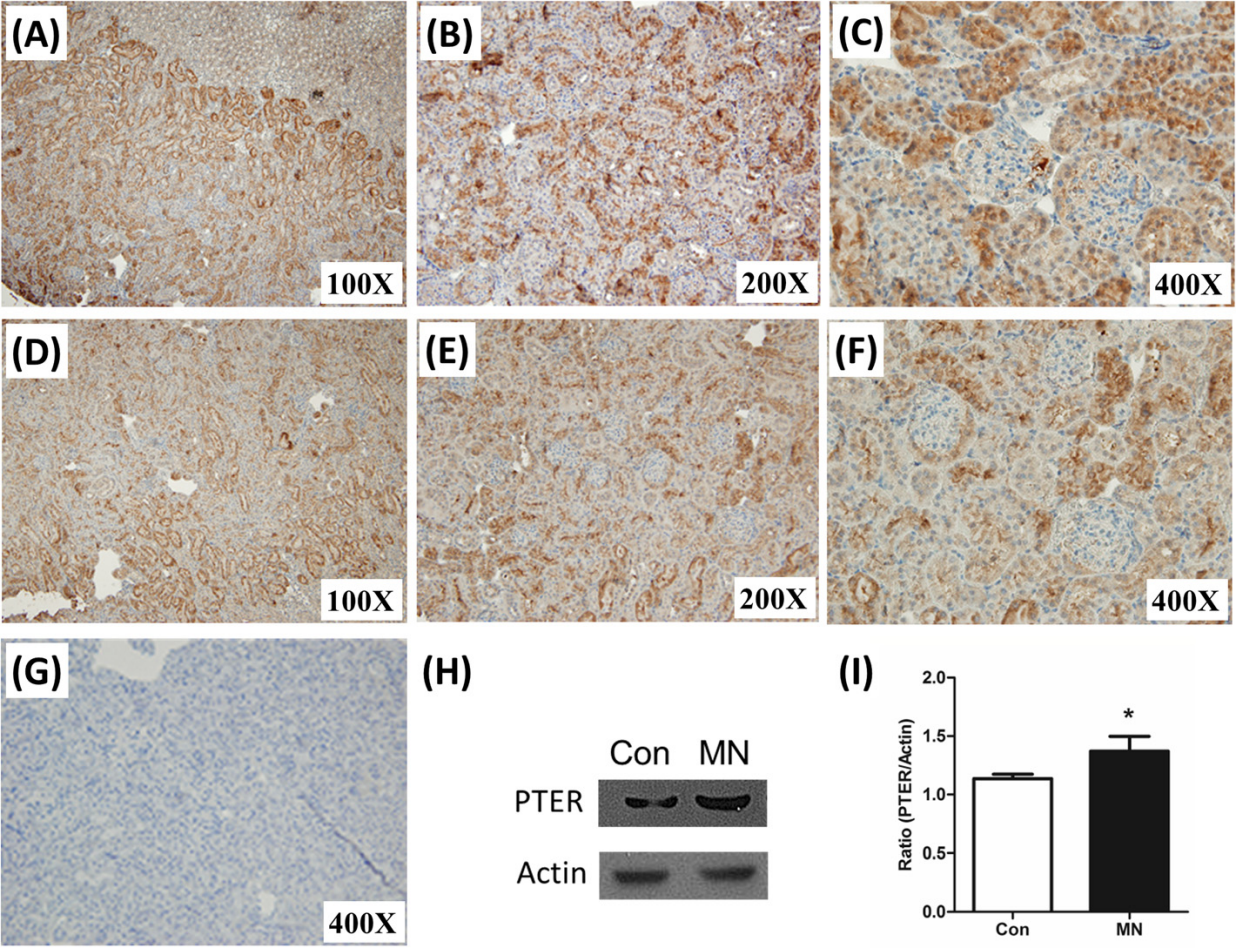
**Differential PTER expression level in the MN mouse model.** At the end of the experiment, kidney samples were collected for histopathological examination and immunoblot analysis. Representative sections from renal tissue in MN **(A, B and C)** and NC **(D, E and F)** groups were examined with PTER immunohistochemical stain, original magnifications: 100× **(A and D)**, 200× **(B and E)** and 400× **(C and F)**. In addition, **(G)** represented negative controls staining without primary antibody in NC section, 400X. Total protein lysate was extracted from the renal cortical region and separated in 10% SDS page by electrophoresis, and further blotting by anti-PTER and β-actin antibodies **(H)**. The bar chart represents the semi-quantification level of the expression density **(I)** (PTER/β-actin). *, p < 0.05.

**Figure 2:**
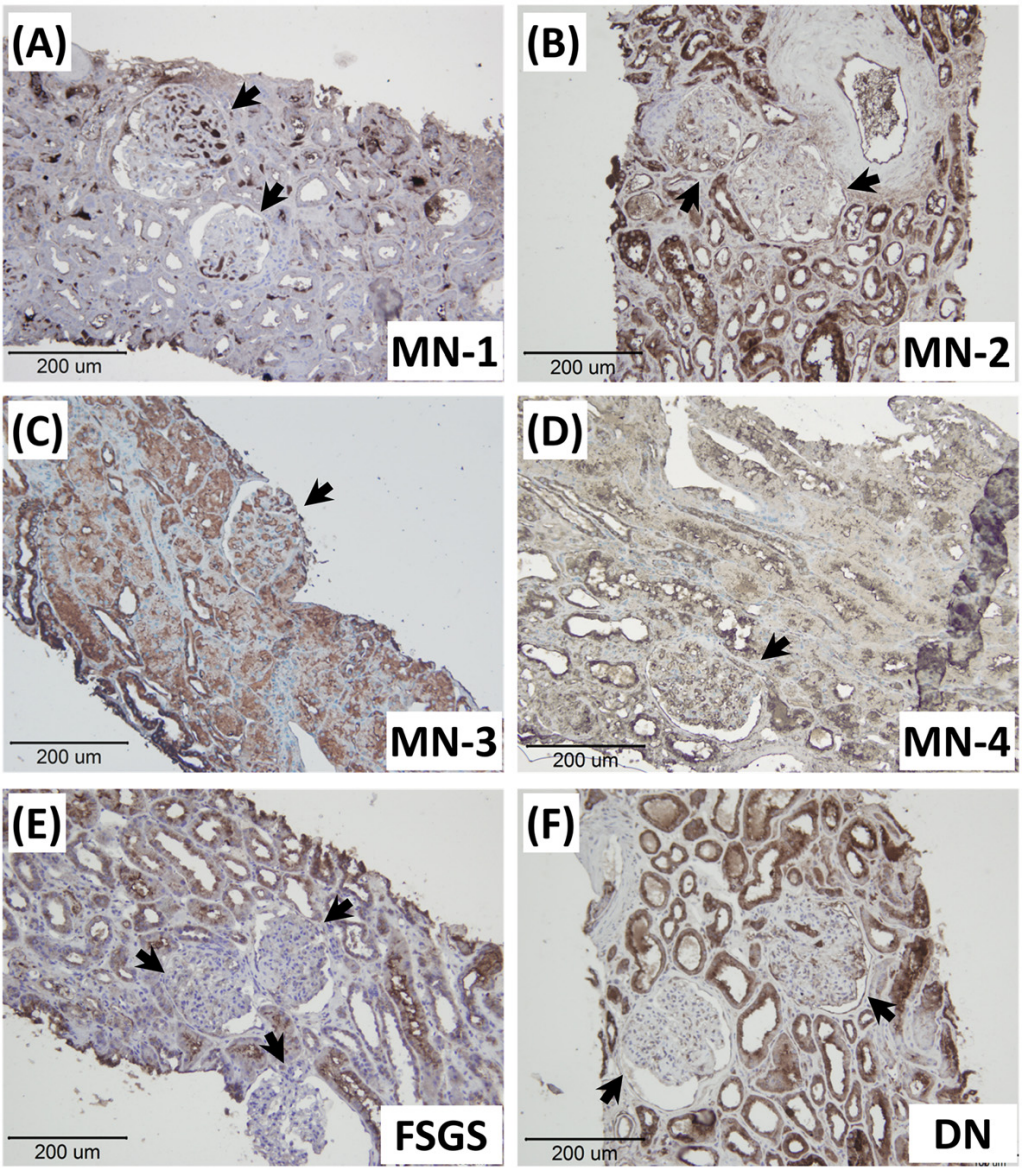
**Expression of PTER in human clinical specimens.** Representative sections from clinical kidney tissue specimens of MN **(A-D)**, FSGS **(E)** and DN **(F)** patients were performed with PTER immunohistochemical stain (arrows indicate glomerulus), original magnifications: 100×. The clinical data are presented in Table 1.

To confirm the up-regulation of PTER protein level in MN, the renal cortical proteins were extracted and analyzed by western blotting. When compared to the control group, the expression of PTER protein was significantly increased in the MN group. Immunohistochemistry stain was also used to analyze the renal distribution pattern and expression level of PTER in MN. PTER protein was expressed in the renal tubular cells in the control group and increased markedly in the full-blown MN, as represented by the marked albuminuria (Figure 1). PTER also strongly expressed in the human MN, DN, FSGS cases, which all presented with marked albuminuria (Table 1 & Figure 2). Table 1 showed all clinical cases’ basic data and tubular expression of PTER. Regarding intensity of PTER in renal tubules, histological pictures were evaluated by two separated pathologists, and scored by grade from + to ++++. The average score was shown in Table 1.

**Table 1:** Clinical data and tubular expression of PTER for patients with MN, FSGS and DN

**Case**	**MN**				**FSGS**	**DN**
	**1**	**2**	**3**	**4**		
Age(y/o)	80	85	61	50	34	47
Gender	M	M	M	M	M	F
U_p_/Ucr	4.1	19.2	3.2	18.9	2.2	2.5
S_cr_(mg/dl)	0.7	3.5	0.8	1.0	1.0	0.8
S_alb_(g/dl)	1.8	1.9	2.6	1.8	2.2	2.8
Intensity of PTER in renal tubules	+	+++	++	++	++	++

Abbreviations: *MN* membranous nephropathy, *FSGS* focal segmental glomerulosclerosis, *DN* diabetic nephropathy, *PTER* phosphotriesterase-related protein, *y*/*o* years old, *M* male, *F* female, *U*_*p*_ urine protein, *U*_*cr*_ urine creatinine, *S*_*cr*_ serum creatinine, *S*_*alb*_ serum albumin.

### Increased PTER mRNA and protein expression in an in vitro albuminuria model

**Figure 3:**
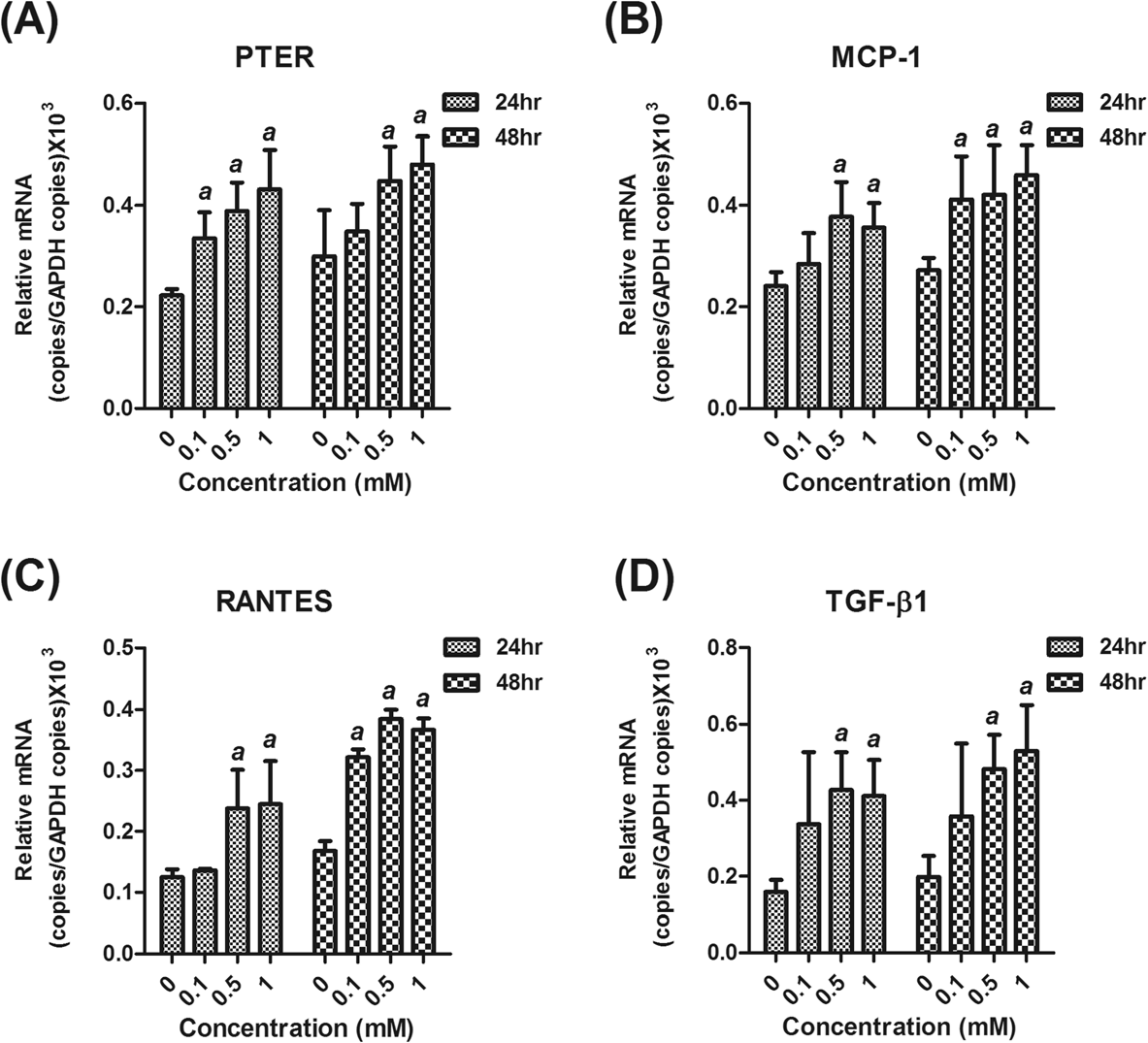
**Increased PTER, MCP-1, RANTES and TGF-β1 expression in an *in vitro *albuminuria model.** HK-2 cells were treated with 1 mM HSA, and the total cellular RNA was isolated at different time points as described in Methods, and subjected to real-time PCR analysis for PTER **(A)**, MCP-1 **(B)**, RANTES **(C)** and TGF-β1 **(D)** expression. The mRNA expression levels were normalized to GAPDH, and the results are presented as mean ± SD. *a*, *p* < 0.05 in comparison with the control group.

**Figure 4:**
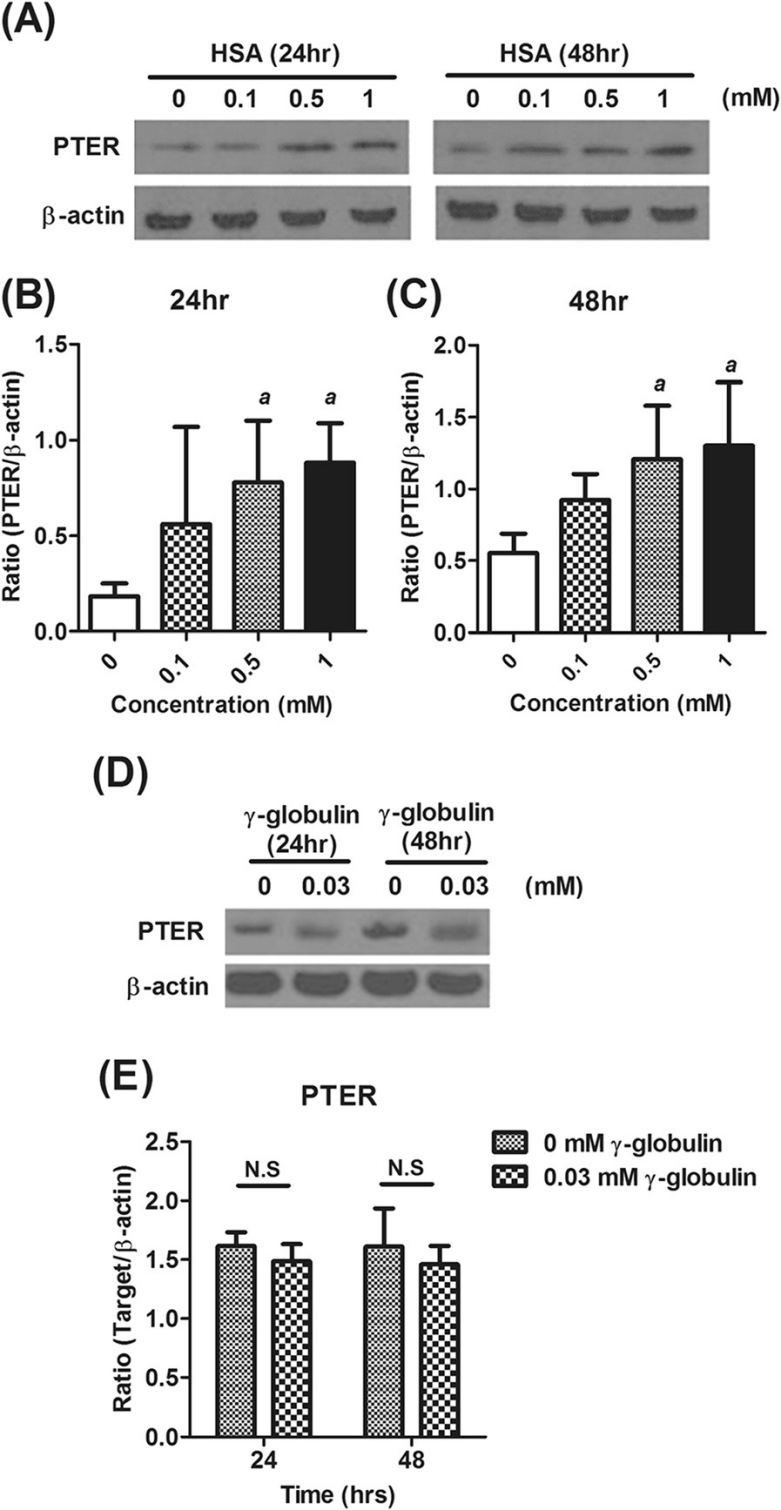
**PTER protein was specifically expressed in response to HSA-induced *in vitro *albuminuria model.** Immunoblot analysis for the expression of PTER at 24 and 48 h in different concentrations of HSA (0.1, 0.5 and 1 mM)-treated HK-2 cells were examined **(A)**. Data represent one out of three experiments. The relative PTER protein levels at 24 **(B)** and 48 h **(C)** were then normalized to β-actin and the results were presented as mean ± SD. *a*, p < 0.05 in comparison with the control group. The expression of PTER at 24 and 48 h in 0.03 mM γ-globulin-treated HK-2 cells were examined **(D)**. The PTER protein expression levels were then normalized to β-actin and the results were presented as mean ± SD **(E)**. *N.S,* No significant difference was found.

**Figure 5:**
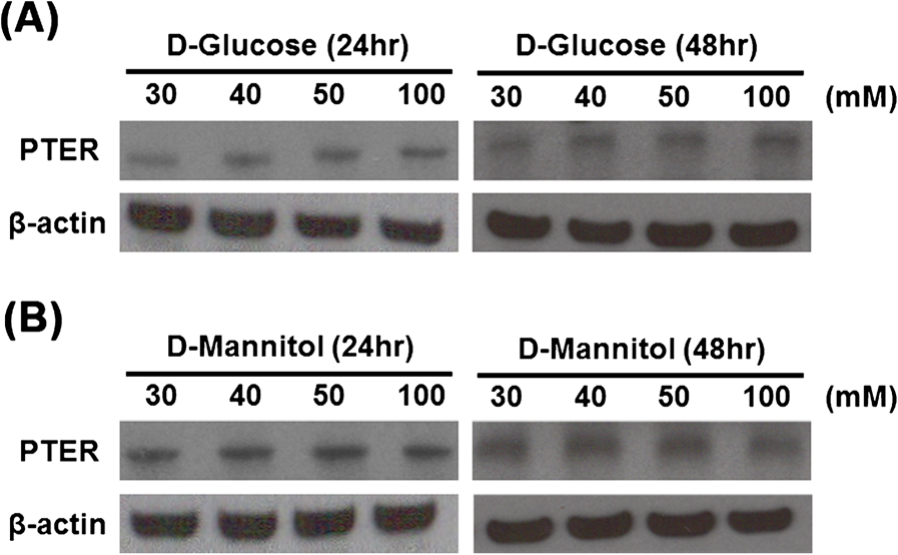
**PTER protein level was not affected by *in vitro *hyperglycemia model.** Immunoblot analysis for the expression of PTER at 24 h and 48 h in different concentrations of D-glucose **(A)** and D-mannitol (30, 40, 50 and 100 mM) were also examined **(B)**. Data represent one out of three experiments.

In order to validate the role of PTER in the pathogenesis of albuminuria, a well-established *in vitro* albuminuria cell model was created by treating immortalized proximal tubular epithelial cell line HK-2 with HSA. HK-2 cells incubated with various concentrations of HSA (ranging from 0.1-1 mM) for 48 h did not show significant alteration in the cell viability (data not shown). Treatment with increased concentrations of HSA had increased the PTER mRNA expression level gradually (Figure 3A). Inflammatory mediators (MCP-1 and RANTES) and pro-fibrotic cytokine (TGF-β1) mRNA had been reported with overexpression in tubular epithelial cells of patients with severe proteinuria and progressive MN [12]. In the *in vitro* albuminuria model, the mRNA expression levels of MCP-1, RANTES and TGF-β1 also increased, representing tubular cell activation (Figure 3B-D). As expected, HSA increased PTER protein expression in time- and dose- dependent manners (Figure 4A-C). However, treatment with 0.03 mM of γ-globulin did not influence PTER protein expression (Figure 4D and E). HK-2 cells were also treated with different levels of glucose and the relative osmolality control mannitol, to further examine the influence of high glucose in PTER protein expression. Unlike HSA treatment, increased glucose level in the culture medium did not affect the PTER protein expression in 24 and 48 h (Figure 5). These data supported our hypothesis that PTER may be specifically participated in response to albuminuria.

### Suppressed PTER expression abrogated albuminuria-induced renal tubular cell activation

**Figure 6:**
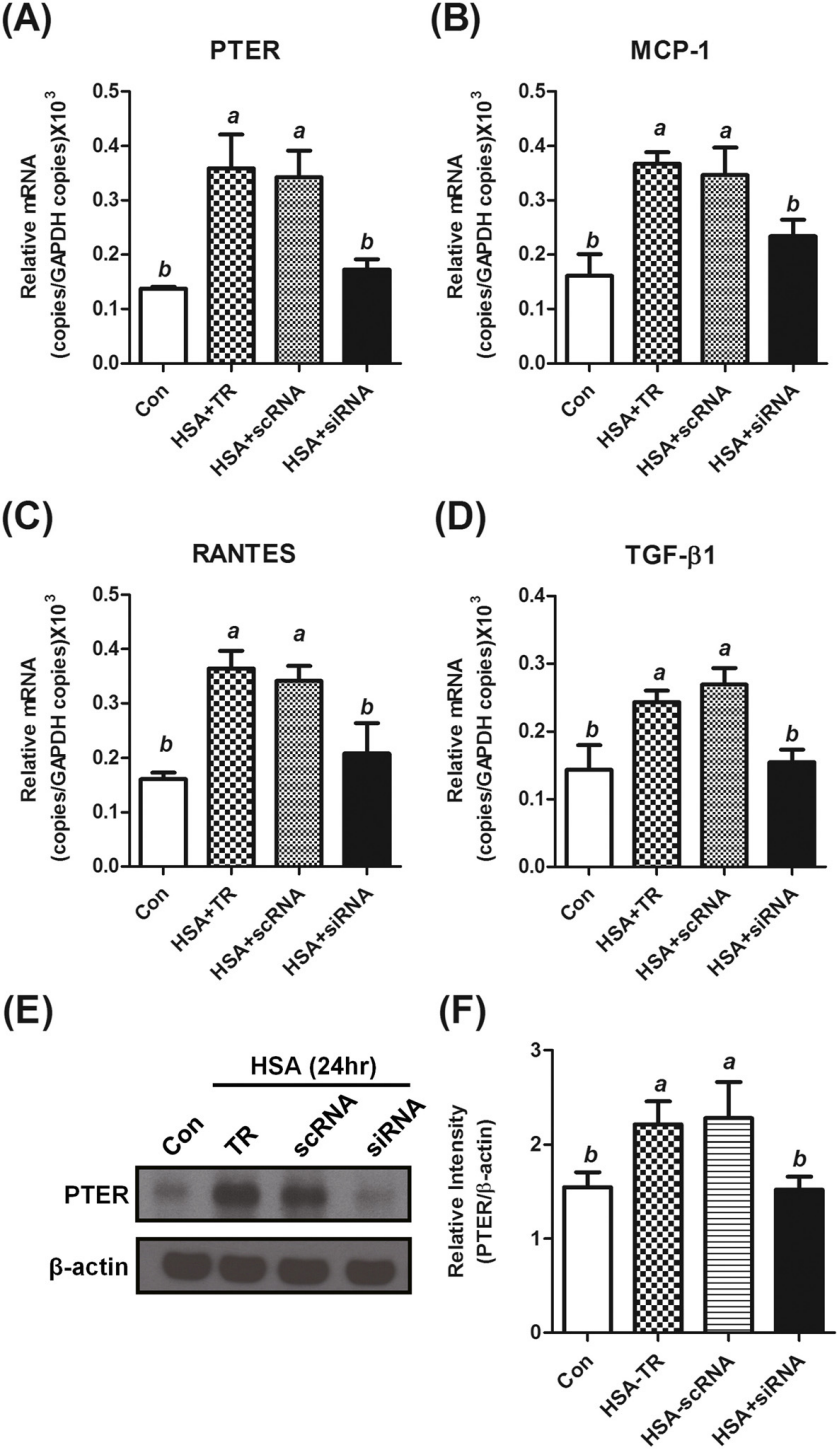
**Silencing PTER expression diminished albuminuria-induced renal tubular cell activation.** The PTER siRNA (siRNA), negative control RNA (scRNA) and transfection reagent alone (TR) transfected HK-2 cells were treated with 1 mM HSA for 24 h for real-time PCR analysis. The mRNA expression levels of PTER **(A)**, MCP-1 **(B)**, RANTES **(C)** and TGF-β1 **(D)** were normalized to GAPDH, and the results are presented as mean ± SD. Total cell protein was extracted at the indicated time points and subjected to analysis of the PTER protein expression levels **(E)**. The relative expression levels were normalized to β-actin and the results were presented as mean ± SD **(F)**. *a*, p < 0.05 in comparison with the control group; *b*, p < 0.05 in comparison with the HSA group.

Specific PTER siRNA was applied to analyze the biological effects of PTER expression in the pathogenesis of albuminuria. Silencing the expression of PTER diminished HSA induced MCP-1, RANTES and TGF-β1 expression levels in HK-2 cell (Figure 6). These data suggest PTER protein participates in the albuminuria-mediated proximal tubular cell activation.

## Discussion

In this study, we identified PTER as being involved in the progression of MN from the animal model and clinical cases. The up-regulation of PTER in the tubular cells may be represented as a sensor protein in response to the albuminuria in the progression of MN. Furthermore, protein overload-induced PTER expression regulates tubular cell activation in the chemokine and pro-fibrotic cytokine expression. These findings also imply that PTER may present a detrimental role in the scenario from MN to ESRD.

Microbial phosphotriesterases are a group of zinc metalloenzymes that catalyze the hydrolysis of one class of highly toxic synthetic compounds known as organophosphates [13]. Originally, the phosphotriesterase was found in the bacteria *Pseudomonas diminuta* and *Flavobacterium* sp [14]. Subsequently, PTER was also isolated and identified in mice and rats. The PTER mRNA is especially expressed in the kidney proximal tubular cells and the expression begins post-natally and lasts to adulthood [11,15]. From the differential cDNA library screening in between normal and cystic kidneys of the C57BL/6 J-*cpk* mouse, PTER was significantly under-expressed in cystic kidneys [11]. The expression of PTER increased in both mRNA and protein levels in the progression of MN and was mainly expressed in the proximal tubular cells (Table 1, Figures 1 and 2). Furthermore, we also suggest that PTER is expressed in FSGS and DN with albuminuria. Taken together, these results suggest PTER may also be involved in some kidney diseases, including MN.

The main manifestation of MN is the presentation of immune complexes deposition along the GBM in the sub-epithelial location, which leads to glomerular capillary wall damage, resulting in albuminuria. In normal conditions, trace amounts of proteins can escape into the glomerular filtrate and be reabsorbed in the apical pore of proximal tubular cells. The process is mediated by the brush border-enriched endocytic receptors megalin and cubilin through ligand-receptor complex formed endocytic vesicles [16-18]. The presence of excessive amounts of urinary proteins may elicit the pathogenesis of chronic tubulointerstitial damage. Urinary protein excretion rate has also been considered as a predictor of ESRD in progressive kidney injury [19]. It has been suggested that megalin may regulate the abnormal protein exposure of tubular cells, activate the transcription factor, and facilitate proinflammatory signaling [20]. However, because megalin can bind the trace amounts of proteins without inducing tubular cell activation; other mechanisms may also be involved in sensing albuminuria. In addition, it has been reported that human γ-globulin (0.03 mM) can be absorbed by opossum kidney epithelial cells through a megalin/cubilin-mediated, clathrin-dependent endocytosis [21]. In the current study, treatment γ-globulin did not induce the PTER expression. This may also suggest that PTER can sense albuminuria, at least in part, but not involved in the megalin-mediated mechanism.

*In vitro* protein overload cell models have been widely used to assess the specific mechanisms linking to excess exposure to proteins [22-24]. In this study, the expression of PTER increased in a concentration- and time-dependent manner in the albumin overload *in vitro* cell model (Figures 3 and 4). It is of note that the PTER expression level was not affected in the excess of glucose or γ-globulin, suggesting its specificity in response to albumin-overload stimuli (Figure 5). The PTER expression pattern is also similar to the expression of MCP-1, RANTES and TGF-β1 (Figure 3); silencing PTER by specific siRNA diminished the expression (Figure 6). These suggest PTER may, at least in part, participate in modulating chemokine and pro-fibrotic cytokine production in mediating renal injury in response to albuminuria. However, the exact mechanism by which PTER mediates renal injury in response to urinary protein requires further study.

## Conclusions

Overall, the findings of this study showed that proximal tubular cells expressing PTER can sense the albumin overload-induced injury and regulate the tubular cell activation. Accordingly, future studies are necessary to discern the detailed mechanisms of PTER in sensing urinary albumin.

## Abbreviations

MN: Membranous nephropathy; ESRD: End-stage renal disease; PTER: Phosphotriesterase-related protein; GBM: Glomerular basement membrane; PLA2R: Phospholipase A2 receptor; VEGF: Vascular endothelial cell growth factor; TSP-1: Thrombospondin-1; K5: Plasminogen kringle domain 5; iTRAQ: Isobaric tag for relative and absolute quantification; cBSA: Cationic bovine serum albumin; HSA: Human serum albumin.

## Competing interests

The authors declare that they have no competing interests.

## Authors’ contributions

CWC, LCC and JSC conceived of the study and designed research. CWC and CJS analyzed data. TLT and LCC performed research. CCW and YFL helped coordinate the study. CWC and JSC wrote the paper. All authors read and approved the final manuscript.
