# Erratum to: Phosphotriesterase-related protein sensed albuminuria and conferred renal tubular cell activation in membranous nephropathy

**DOI:** 10.1186/s12929-016-0252-5

**Published:** 2016-04-04

**Authors:** Chao-Wen Cheng, Li-Chien Chang, Tzu-Ling Tseng, Chia-Chao Wu, Yuh-Feng Lin, Jin-Shuen Chen

**Affiliations:** Graduate Institute of Clinical Medicine, College of Medicine, Taipei Medical University, Taipei, Taiwan; Center for Translational Medicine, College of Medical Science and Technology, Taipei Medical University, Taipei, Taiwan; Graduate Institute of Medical Sciences, Taipei, Taiwan; School of Pharmacy, National Defense Medical Center, Taipei, Taiwan; Biomedical Technology & Device Research Laboratories, Industrial Technology Research Institute, Hsinchu, Taiwan; Division of Nephrology, Department of Internal Medicine, Tri-Service General Hospital, National Defense Medical Center, No. 325, Section 2, Cheng-Kung Road, Neihu 114 Taipei, Taiwan, Republic of China; Department of Internal Medicine, Shuang Ho Hospital, Taipei Medical University, New Taipei, Taiwan

Following publication of the article “Phosphotriesterase-related protein sensed albuminuria and conferred renal tubular cell activation in membranous nephropathy” [1], it has come to our attention that the affiliation number 6 was incorrectly presented as: Division of Nephrology, Department of Internal Medicine, Tri-Service General Hospital, 325, Sec. 2, Cheng-Kung Rd., Neihu 114 Taipei, Taiwan, Republic of China. This should have been presented as: Division of Nephrology, Department of Internal Medicine, Tri-Service General Hospital, National Defense Medical Center, No. 325, Section 2, Cheng-Kung Road, Neihu 114 Taipei, Taiwan, Republic of China. The authors would like to sincerely apologize for any inconvenience or confusion this may have caused.
